# Can Pre-Pregnancy Body Mass Index and Maternal Exercise Affect Birth and Neonatal Outcomes—A Cross Sectional Study

**DOI:** 10.3390/nu15234894

**Published:** 2023-11-23

**Authors:** Anna Weronika Szablewska, Jolanta Wierzba, Rita Santos-Rocha, Anna Szumilewicz

**Affiliations:** 1 Department of Obstetric and Gynaecological Nursing, Institute of Nursing and Midwifery, Medical University of Gdansk, Debinki 7, 80-211 Gdansk, Poland; 2 Department of Pediatric and Internal Medicine Nursing, Institute of Nursing and Midwifery, Medical University of Gdansk, Debinki 7, 80-211 Gdansk, Poland; kwierz@gumed.edu.pl; 3 ESDRM Department of Physical Activity and Health, Sport Sciences School of Rio Maior, Polytechnic Institute of Santarém, 2040-413 Rio Maior, Portugal; ritasantosrocha@esdrm.ipsantarem.pt; 4 CIPER Interdisciplinary Centre for the Study of Human Performance, Faculty of Human Kinetics (FMH), University of Lisbon, 1495-751 Lisbon, Portugal; 5 Department of Physical Culture, Gdansk University of Physical Education and Sport, 80-336 Gdansk, Poland; anna.szumilewicz@awf.gda.pl

**Keywords:** preterm birth, pre-pregnancy BMI, exercise, birth outcomes

## Abstract

There has been a dramatic worldwide increase in the prevalence of obesity or overweight and physical inactivity in women of reproductive age. Growing evidence suggests that pre-pregnancy maternal abnormal body mass index (BMI) and lower physical activity level are associated with poor maternal health and perinatal outcomes. The aim of this study was to assess how self-perceived exercise and pre-pregnancy BMI are associated with preterm birth, low birth weight, and type of birth. We conducted a retrospective cross-sectional study of 394 Polish women in the postpartum period. We used a questionnaire with the structure of the medical interview. To analyze factors related to birth outcomes, we used the Pearson’s Chi-squared test of independence and odds ratio (OR), with a corresponding 95% confidence interval (CI), followed by a multiple logistic regression. Women who reported being physically active before pregnancy (*p* = 0.00) and during pregnancy (*p* = 0.03) were more likely to give birth on time and had a lower incidence of very-premature and extremely premature births compared to inactive women. Importantly, they were more likely to have vaginal birth (*p* = 0.03). Pre-pregnancy BMI influenced the week of delivery, i.e., inadequate, too-high BMI contributed to an increase in the percentage of premature births [OR (95% CI) = 1.19 (1.06; 1.34)]. The findings indicate that promoting physical activity and weight management remains a priority in public health policy, and women of childbearing age should be encouraged to adopt or maintain an active and healthy lifestyle during pregnancy in order to avoid sedentary- and obesity-associated risks affecting birth and newborns’ health.

## 1. Introduction

The recent data have shown that regular physical activity during pregnancy and before pregnancy has a positive effect on the physical and psychological condition of the future mother, fetal development, parturition, and functioning during the postpartum period [1,2,3,4,5,6]. What is more, physical activity started in pregnancy may have impact on a lifelong change to a health-promoting lifestyle. Research has also proved that the prenatal physical activity of mothers has a long-term effect on the health of the children, including a reduction in the risk of obesity in later life [7,8].

The presence of abnormal body weight before pregnancy and in the first trimester of pregnancy is a crucial risk factor of preterm birth (PTB) and low birth weight (LBW) [9,10]. The global preterm birth rate has remained constant for over 20 years, amounting to 9.6–11% [11,12,13]. On the other hand, data from the World Health Organization (WHO) show that in recent years an increase in the number of premature births has been observed in many countries (mainly in industrialized countries). Prematurity and low birth weight are one of the most important and still valid challenges of modern medicine, and are also a socio-economic problem. It is important to emphasize that the incidence of preterm birth is a multi-faceted and multifactorial aspect. Its occurrence is influenced by maternal, fetal, genetic, infectious, and environmental factors. In many cases, the cause of preterm birth remains unknown, making it an unresolved and still current problem in perinatology [14]. It is also important to note that the factors determining the incidence of preterm births change over the years and vary according to geographical location [15]. Preterm babies require interdisciplinary, specialist treatment, diagnostics, and rehabilitation, sometimes lasting their entire life. The literature data show that approximately 15 million premature babies are born in the world each year [11,16,17].

In many studies, researchers have mentioned that abnormal pre-pregnancy BMI is a very important independent risk factor of PTB. This value also determines the recommended maternity weight gain during pregnancy. The data obtained in this way are considered the most important indicators of the nutritional status of a pregnant woman [18]. Abnormal BMI is also associated with an increased risk of gestational diabetes, pre-eclampsia and eclampsia, pregnancy-induced hypertension (PIH), and other perinatal abnormalities [19,20,21]. The consequences of the increase in the frequency of premature childbirths and low birth weight have led to a critical analysis of the factors that may affect their occurrence and effects (social, medical, psychological, and economic). Focusing on a specific population of Polish women adds a unique dimension to this field. By delving into a distinct demographic, our research contributes to a better understanding of the relationship between physical activity, BMI, and birth outcomes within the context of the Polish population. Additional exploration of how pre-pregnancy BMI influences the week of pregnancy completion (using categories), particularly the association with an increased percentage of premature births (which is still an unsolved problem), provides valuable insights.

### Aim

The aim of this study was to assess how pre-pregnancy BMI and self -perceived maternal exercise are associated with preterm birth, low birth weight, and other newborn and birth outcomes.

## 2. Materials and Methods

### 2.1. Study Design

The present study was a multi-center, retrospective, cross-sectional study among a group of 394 Polish women (in the Polish population, ethnic minorities represent a relatively small percentage of the total population, so we did not distinguish ethnic groups) in the postpartum period and after singleton pregnancy; of which, 153 women delivered preterm (22–37 weeks) (38.8%) and 241 had a full-term birth (38–42 weeks) (61.2%). We used a questionnaire with the structure of the medical interview. Also, medical history, especially maternal and neonatal outcomes, were analyzed (see the Supplementary Materials). We followed the STROBE guidelines for cross-sectional studies [22]. All procedures were performed in accordance with the principles outlined in the Declaration of Helsinki of the World Medical Association (WMA) and approved by the Bioethics Commission of the Gdansk Medical University, no. NKBB/393/2015 for studies involving humans.

### 2.2. Setting

The study was conducted in 2 hospitals of tertiary referral centers (the Poland classification system establishes levels of maternal care that pertain to level I of perinatal care: the care of a physiologically progressing pregnancy, labor and the puerperium, and a healthy newborn (possibly short-term pregnancy pathology); level II of perinatal care: the care of intermediate-level pathological pregnancies; and level III of perinatal care: the care of the most severe pregnancy pathology) in the northern region of Poland. The period of data collection and patient eligibility was from 1 January 2016 to 1 January 2018.

### 2.3. Participants

All participants were informed of the study objectives and provided their voluntary consent to participate, by marking consent to participate in the survey in the questionnaire and consent for access to medical records (it was one of the inclusion criteria). The parents were informed that taking part in the research would not affect their child’s diagnostic and therapeutic process and that the child would not be exposed to additional procedures. The children’s mothers, as their legal carers, also consented to access to the documentation concerning the newborn child.

The principal investigator had a personal conversation with each mother of the children, explaining all concerns about the conduct of the study and the recruitment process. Mothers were invited to the study during their stay in the maternity ward after delivery or during a visit to the newborn in the neonatal intensive care unit (if the baby’s mother was not invited to participate after delivery and the baby’s condition required prolonged hospitalization). The children’s parents had the right to ask questions. Each child and mother included in the study were given an identification number, based on data from the medical records (such as mode of pregnancy completion, duration of pregnancy, maternal weight gain during pregnancy, the newborn’s birth weight and Apgar score, anthropometric data of the mother before pregnancy) that were completed, to minimize information bias.

The study included 394 mothers aged 19 to 44 years (hospitalized in the maternity wards of hospitals) and their children born, alive, between 22 and 42 weeks’ gestation, who met the inclusion criteria (hospitalized in the neonatology and neonatal intensive care unit of the associated units or in their mothers room in the system).

**Figure 1:**
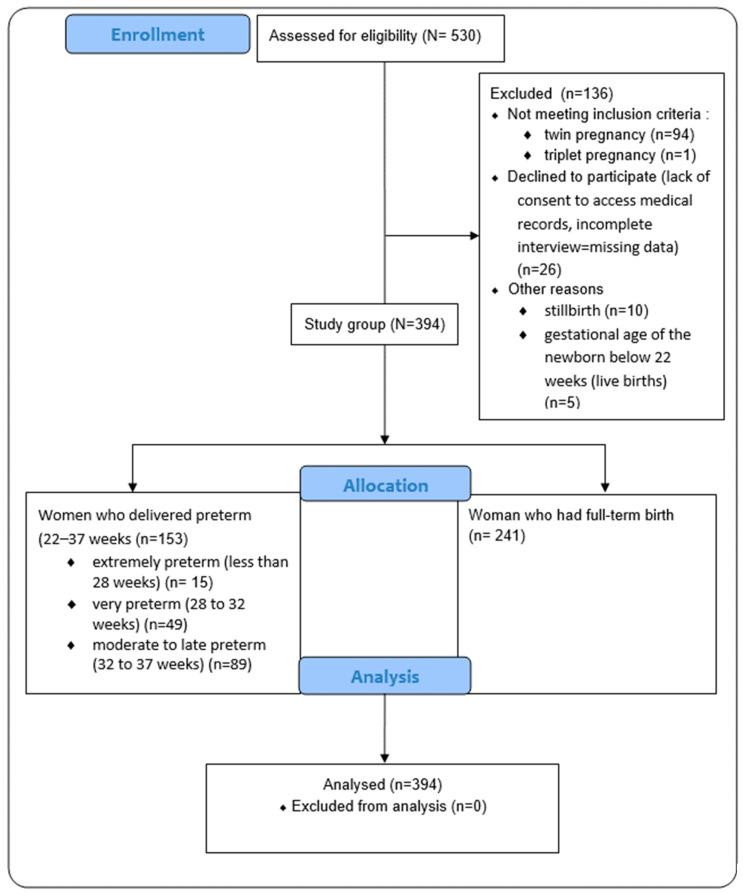
The flow of participants through the study.

Approximately 5000 mothers received an invitation to take part in the study. Only 10% responded, and 136 participants were subsequently excluded from the study due to not meeting the eligibility criteria (as a result the rate of preterm birth was 38.8%). We conducted the study at the level III reference medical centers, where the rate of premature births was assumed to be higher than in other hospitals, due to the access to specialized equipment. Nevertheless, for our study, this increased rate of preterm birth compared to the general population appeared to be beneficial, providing access to a larger group of newborns born prematurely. This allowed for more accurate statistical analysis and more accurate inferences. The participants’ flow through the study is presented in Figure 1.

### 2.4. Inclusion and Exclusion Criteria

The inclusion criteria were mothers having a single, alive birth after 22 weeks of pregnancy, those that gave birth at the public hospitals, and those understanding the Polish language.

We excluded multiple gestations (assuming that it could be a potential reason for preterm birth and low birth weight), lack of consent to access medical records, obtaining an incomplete interview questionnaire, death of a newborn in the perinatal period, stillbirth, and gestational age of the newborn below 22 weeks (in the case of alive births).

### 2.5. Variables and Data Sources

#### 2.5.1. Maternal Variables

Understanding the importance of pre-pregnancy exercise on the pregnancy and newborn outcomes, we included two subcategories of the study women in terms of their exercise habits:

Self-perceived exercise before pregnancy—exercise performed by the mother 6 months before becoming pregnant, excluding normal home- and work-related activity, but including active transportation, and autonomous and supervised physical exercise. As “exercise” we considered all forms of physical activity that were planned, structured, repetitive, and performed with the goal of improving health or fitness [23]. We noted these data from the survey addressed to mothers. This variable was categorized as YES or NO.

Self-perceived exercise during pregnancy (new exercise, started after becoming pregnant)—exercise started after becoming pregnant and performed by the mother during pregnancy, excluding normal home and work-related activity, but including active transportation, and autonomous and supervised physical exercise. We noted these data from survey addressed to mothers. This variable was categorized as YES or NO.

Pre-pregnancy BMI category—this value was calculated by taking a mother’s weight (before becoming pregnant or in the first weeks of pregnancy-data obtained from pregnancy chart), in kilograms, divided by their height, in meters squared, or BMI = weight (in kg)/height^2^ (in m^2^). The number generated from this equation was marked as the individual’s pp-BMI number. These classifications for BMI are in use by the National Institute of Health (NIH) and the World Health Organization (WHO) for White, Hispanic, and Black individuals [24]. The BMI number and classifications are listed below:

- Overweight and obesity—BMI greater than or equal to 25.0 and greater than 30 kg/m^2^;
- Normal weight—BMI greater than or equal to 18.5 to 24.9 kg/m^2^;
- Underweight—BMI under >18.5 kg/m^2^;

We connected these two categories (overweight and obesity) because of the small group of obese mothers.

Maternal weight gain (WG) (kg)—this was calculated by recommendations for total and rate of Weight Gain During Pregnancy, by Pre-pregnancy Body Mass Index (pp-BMI), data from the Institute of Medicine/National Research Council [25]:

- Overweight and obesity (25.0–29.9 and 30.0 or higher)—7.0–11.5 and 5.0–9.0 kg
- Normal weight (18.5–24.9)—11.5–16.0 kg
- Underweight (less than 18.5)—12.5–18.0 kg

Methods of labor—the method of pregnancy completion was recorded from the participants’ medical records. Vaginal delivery, caesarean section, or operative delivery (vacuum, forceps delivery) were considered. Perineal episiotomy and degrees of perineal tears were not analyzed in cases of vaginal delivery.

#### 2.5.2. Newborn Variables

Gestational age at delivery (number of weeks category)—the newborn’s age at birth, determined by the week of gestation according to Neagele’s rule or, if there was a discrepancy between the gestation date and the first-trimester ultrasound, according to the first-trimester ultrasound. The information on the week of pregnancy completion was recorded from the medical records and verified by the medical staff.

Particular consideration was given to completion of the pregnancy prematurely (PTB, preterm birth), i.e., between 22 and 27 weeks’ gestation. The subcategories of preterm births, according to the WHO, by gestational age were used [26,27]:

- Extremely preterm birth (less than 28 weeks);
- Very preterm birth (between 28 and 32 weeks);
- Moderate and late preterm birth (32 to 37 weeks).

The proposed subdivision was taken into account due to the different proportion of preterm births in each category, with the largest group being late preterm births, which also has an impact on the prognosis and further development of the newborn. It is also important to underline here that the gestational age at delivery categories were related to the referral of the patient to the appropriate reference center (hospital reference levels), which is crucial issue in planning the care of pregnant women.

Newborn’s birth weight (in grams)—the birth weight of the neonate is an important clinical criterion. Many times, the birth weight of a neonate born prematurely corresponds to that of a full-term neonate, i.e., between 2500 g and 4000 g. It also happens that a neonate born on time is born with a low birth weight, which can be indicative of hypotrophy. We noted these data from medical records, and qualified newborns for subcategories of newborn weight [28].

Division of neonates by birth weight:

- Hypertrophic neonate, too large for gestational age (LGA, large for gestational age), defined as macrosomia-neonatal weight above 4000 g;
- Eutrophic neonate, weight appropriate for gestational age (AGA, appropriate for gestational age)—body weight between 2500 g and 4000 g;
- Hypotrophic neonate, too small for gestational age (SGA)—body weight below the 10th centile for gestational age or less than 2500 g;
- Low birth weight (LBW) neonate—weight between 2500 g and 1500 g;
- Very-low birth weight newborn (VLBW)—weight between 1499 g and 1000 g;
- Extremely low birth weight (ELBW)—weight between 1000 g and 750 g;
- Incredibly low birth weight newborn (ILBW, or incredibly low birth weight)—weight less than 749 g.

Preterm birth is the leading cause of low birth weight in both developing and highly developed countries. These pathologies (LBW and PTL) most often occur together. In our work we described normal weight and compared the results with LGA, LBW, VLBW, ELBW, and ILBW because of the potential negative influence of this factor on the babies; according to previous studies, they were more likely to die during their first month of life and those who survived face lifelong consequences, including a higher risk of stunted growth, lower IQ, and adult-onset chronic conditions such as obesity, diabetes, and cardiovascular disease (CVD) [28].

Apgar score in 1st minute (0–10 points)—As an indicator of the general condition immediately after birth, the Apgar scale was chosen as a universal and easily obtainable parameter [29] from the records (given that the clinical material and the analysis of the records concerned different institutions, and the analysis was intended to compare neonates born full-term and prematurely). We noted these data from newborn medical records.

### 2.6. Statistical Analysis

The data were analyzed using statistical software: the Statistica 12.0 version (advanced package) and PQStat v 1.8.0.476. Pearson’s Chi-squared test of independence was used to present the differences in the rates of full term vs. preterm deliveries and the differences in the birth weight categories in subgroups of active and inactive women. With the same test, we analyzed the rates of various modes of delivery in subgroups of women with the different pre-pregnancy BMI. Logistic regression analysis was performed (analyzed variables: group; mother’s weight at pregnancy; mother’s pp-BMI; weight gain in pregnancy). Multiple regression analysis was performed in order to analyze the strength of the relationship between maternal BMI and newborn variables (newborn’ birth weight, Apgar score). The level of significance adopted was 0.05. Sample size was computed using G-power 3.1. (correlations: two independent Pearson, effect size q = 0.5; α error of probability *p* = 0.05; power = 0.95; allocation ratio N2/N1 = 2; result: sample size group 1 = 68; sample size group 2 = 135; total sample size = 203, using variable: maternal exercise before pregnancy). The calculated sample size was smaller than our final study group; however, due to access to more women after childbirth, we decided to analyze larger data, which in turn allowed for subgroup analysis.

## 3. Results

A total of 394 women participated in the study: 153 women who delivered preterm (22–37 weeks-study group) and 241 who had a full-term birth (38–42 weeks-control group). Demographic characteristics, maternal data, and pregnancy outcomes are shown in Table 1 and Table 2.

We found that 25.1% women with normal weight (BMI 18.5 to 24.9) had EWG, excessive gestational weight gain; 40.0% had AWG, appropriate weight gain; and 34.9% LWG, too little weight gain (recommended WG 11.5–16.0 kg). Women who were overweight and obesity (BMI 25.0 to <30) had EWG in 62.0%, AWG in 20.6%, and LWG in 17.4% (recommended WG 7.0–11.5 kg). Women who were underweight (>18.5) had EWG in 5.5%, AWG in 66.7%, and LWG in 27.8% (recommended WG 12.5–18.0 kg).

**Figure 2:**
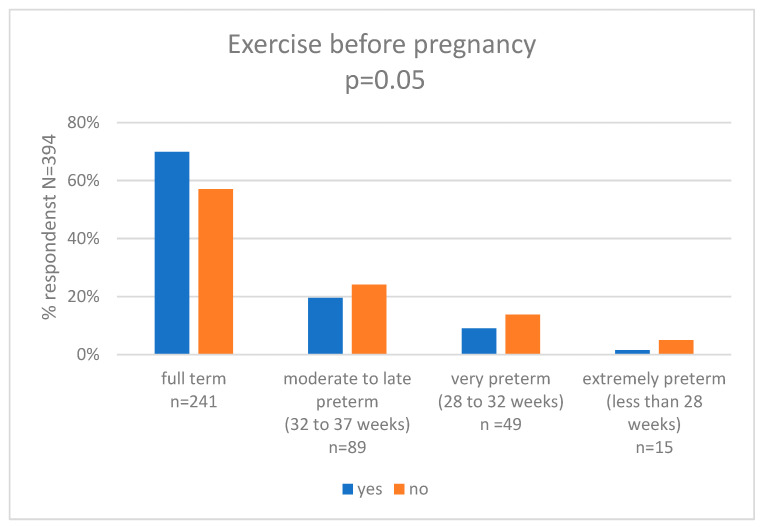
Exercise before pregnancy and gestational age at delivery (weeks).

In our research, we assessed self-perceived exercise before pregnancy and we compared the results with the week of pregnancy category. Chi-squared Pearson’s test showed statistically significant differences between the groups (*p* = 0.05). In the group of women who were active, 69.9% (*n* = 93) delivered full term, 19.5% (*n* = 26) had moderate to late preterm birth, 9.0% (*n* = 12) had very preterm birth, and 1.4% (*n* = 1) had extremely preterm birth. In the group of women who were not active, 56.7% (*n* = 148) delivered full term, 24.1% (*n* = 63) had moderate to late preterm birth, 14.2% (*n* = 37) had very preterm birth, and 5.0% (*n* = 13) had extremely preterm birth. The results are shown in Figure 2 and Supplementary Table S7.

**Figure 3:**
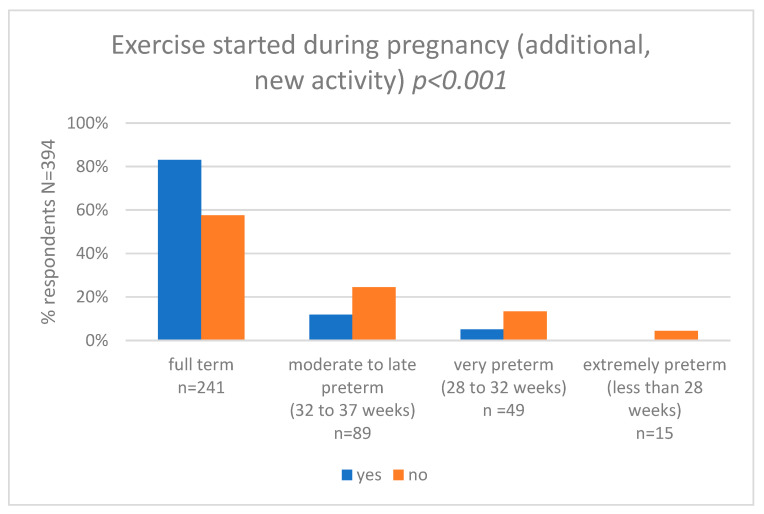
Exercise started during pregnancy and gestational age at delivery (weeks).

We also asked women about self-perceived maternal exercise during pregnancy (new exercise, started after becoming pregnant) and we compared the results with that of the gestational age at delivery (weeks) category. Pearson’s chi-squared test showed statistically significant differences between the groups (*p* < 0.001). In the group of women who started to be active during pregnancy, 83.0% (*n* = 50) delivered full term, 11.9% (*n* = 7) had moderate to late preterm birth, 5.1% (*n* = 3) had very preterm birth, and 0.0% (*n* = 0) had extremely preterm birth. In the group of women who were not active, 57.6% (*n* = 192) delivered full term, 24.5% (*n* = 82) had moderate to late preterm birth, 13.4% (*n* = 46) had very preterm birth, and 4.5% (*n* = 15) had extremely preterm birth. The results are shown in Figure 3 and Supplementary Table S8.

**Figure 4:**
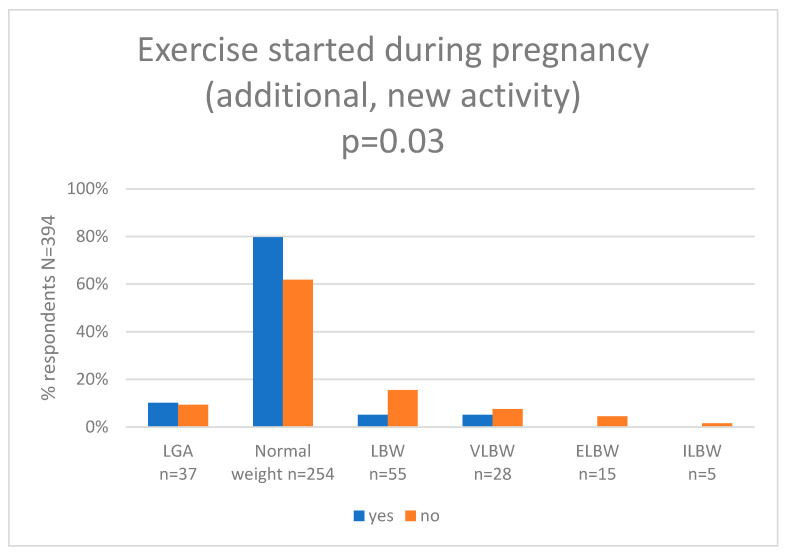
Exercise started during pregnancy and newborn birth weight (g). (LGA—large for gestational age (more than 4000 g), LBW—low birth weight (less than 2500 g), VLBW—very-low birth weight (less than 1500 g), ILBW—incredibly low birth weight (less than 750 g)).

In the next step, we assessed self-perceived maternal exercise during pregnancy and we compared results with the newborn birth weight category. Pearson’s chi-squared test showed statistically significant differences between the groups (*p* = 0.03). In the group of women who were active, 10.2% (*n* = 6) newborns had LGA, 79.6% (*n* = 47) newborns had a normal birth weight, 5.1% (*n* = 3) had low birth weight (LBW), 5.1% (*n* = 3) had very-low birth weight (VLBW), 0.0% (*n* = 0) had extremely (ELBW), and 0.0% (*n* = 0) had incredible low birth weight (ILBW). In the group of women who were not active, 9.2% (*n* = 31) newborns had LGA, 61.8% (*n* = 207) newborns had a normal birth weight, 15.5% (*n* = 52) had low birth weight (LBW), 7.5% (*n* = 25) had very-low birth weight (VLBW), 4.5% (*n* = 15) had extremely (ELBW), and 1.5% (*n* = 5) had incredible low birth weight (ILBW). The results are shown in Figure 4 and Supplementary Table S9.

**Figure 5:**
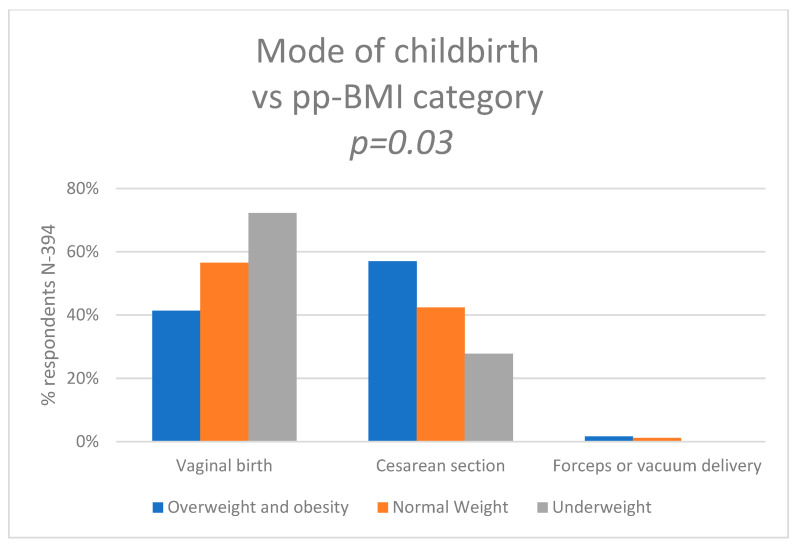
Mode of the childbirth vs. pre-pregnancy BMI category.

We also wanted to know how pre-pregnancy BMI affected the mode of birth. Pearson’s chi-squared test showed statistically significant differences between the groups (*p* = 0.03). Of the women who had normal weight before pregnancy, 56.5% (*n* = 144) had a VB, 42.3% (*n* = 108) had a CS, and 1.2% (*n* = 3) had forceps or vacuum delivery. Of the women who were obese or overweight, 41.3% (*n* = 50) had VB, 57.0% (*n* = 69) had CS. and 1.7% (*n* = 2) had an assisted vaginal birth. Of the mothers who were underweight, 72.2% (*n* = 13) had VB and 27.8% (*n* = 5) had a CS or had an instrumental delivery. The data are shown in Figure 5 and Supplementary Table S10.

The variables were collected on an interval scale. On the basis of the knowledge gained after running a multivariate regression model simulation of the various selected variables, we planned to predict how the duration of pregnancy is related singly (week of delivery) with the specified variables during this simulation. Based on the interpretation of the obtained results, we can assume that some of the variables do not have a significant impact on the gain and may be redundant.

We built a multiple linear regression model by selecting the following variables: pp-BMI (pp-BMI), newborn’s birth weight (NBW), and APGAR (1st minute). As a result, the coefficients of the regression equation and measures were calculated to assess the quality of the model. Based on the estimated value of the b-factor, the relationship between week of pregnancy completion (WPC) and all the independent variables can be described with the equation:

WPC = 26.366 − 0.062.56 (pp-BMI) + 0.003 (NBW) + 0.531 (APGAR) + [1.962]

The model fits well, as evidenced by a small standard error estimate SE = 1.96; high value of the coefficient multiple determination R^2^ = 0.780825; corrected coefficient multiple determination R^2^adj = 0.78; and the result of the F test of the analysis of variance: *p* < 0.000.

Interpretation of the model: if the mother’s pp-BMI increases by 10 kg/m^2^, the time to complete pregnancy will be shortened by an average of 4 days, assuming ceteris paribus. If the pregnancy is lengthened by 3.5 days, the Apgar index increases by 1 point, assuming ceteris paribus. If the time to complete the pregnancy increases by 3 weeks, the weight of the child at birth increases by 1000 g, assuming ceteris paribus. Additionally, 78.08% of the total variability in the time of completion of pregnancy was explained by the model (interpretation for the R^2^ index value). Note that the multivariate regression equation only applies to the study population under unchanged conditions (ceteris paribus).

The fit quality of the model was not high (R^2^Pseudo = 0.03, R^2^Nagelkerke = 0.05, and R^2^Coxa − Snella = 0.04). At the same time, the model was statistically significant (*p* = 0.001 of the likelihood-ratio test) and, therefore, the independent variables in the model were statistically significant. The Hosmer–Lemeshow test result indicates no statistical significance (*p* = 0.09). However, in the case of the Hosmer–Lemeshow test, the lack of significance is desirable because it indicates the similarity of the size of the observed groups and the predicted probability.

The risk of premature delivery depends on the above-mentioned variables as described by the OR: variable maternal BMI before pregnancy OR [95% CI] = 1.19 [1.06; 1.34]. The risk of preterm labor increases with increasing maternal BMI before pregnancy.

**Figure 6:**
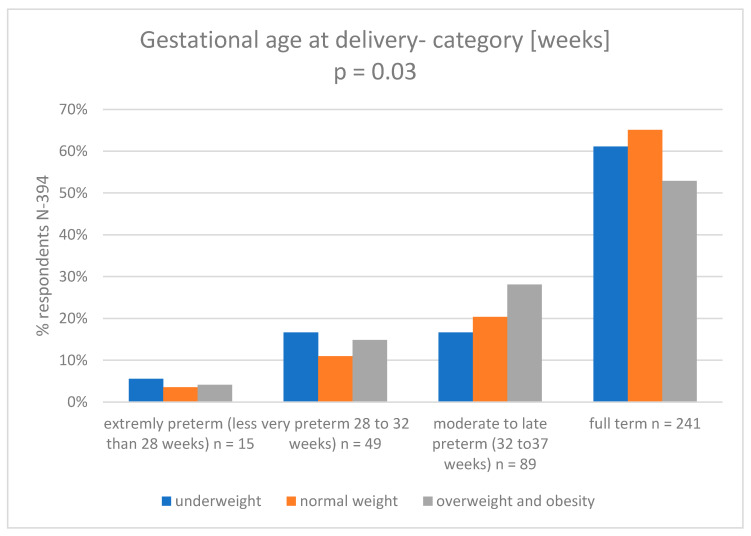
Gestational age at delivery category (weeks) vs. maternal pp-BMI4.

In the Supplementary Materials (Supplementary Tables S2–S6 and S11) we present a logistic regression model (analyzed variables: group; mother’s weight at pregnancy; mother’s pp-BMI; weight gain in pregnancy). In the Figure 6 we presented categories of gestational age at delivery vs maternal pp-BMI.

## 4. Discussion

The principal objective of this study was to identify the associations of self-perceived maternal exercise undertaken before and during pregnancy, pp-BMI, and selected birth and newborn’s outcomes.

The most important findings of this work are that maternal exercise before and during pregnancy and normal maternal pp-BMI can positively affect birth outcomes, leading to a reduced risk of PTB and births of children with low birth weight.

Worldwide, there has been a dramatic increase in the prevalence of obesity and overweight in women of reproductive age. Growing evidence suggests that abnormal pre-pregnancy body mass index (pp-BMI) is associated with poor maternal and perinatal outcomes [19,30]. Proper diet and physical activity have the potential to reduce weight gain and alter pregnancy outcomes [31]. The effect of these interventions across pregnant women and woman of reproductive age may have implications for clinical management and the provision of care. The association of physical activity before and during pregnancy with birth outcomes needs evaluation using robust data. The lack of information among women of reproductive age about the benefits of the exercise during pregnancy and the low level of social support are two of the factors hindering engagement in prenatal exercise programs. A knowledge of health advantages can lead to more favorable attitudes towards exercise during pregnancy among women, exercise professionals, and healthcare providers [5].

With the increasing amount of scientific evidence on the positive effects of prenatal physical activity, authors from different countries observe its insufficient level in pregnant women [32]. The gestational period is an opportunity to promote positive health behaviors [5,33]. Moreover, in many countries one can find guidelines supporting moderate-intensity physical activity during pregnancy, with specific frequency and duration/time indicated [5,33]. Also, higher intensity exercise programs, which can meet the expectations and needs of very active women before pregnancy, have become more and more popular recently [34]. Nowadays, sufficient evidence is available that shows various types and intensities of maternal exercise enhance proper fetal and child development, including their normal weight gain and metabolism [34,35]. According to researchers, prenatal exercise interventions reduced gestational weight gain and the risk of gestational diabetes for overweight and obese pregnant women, which reinforced the benefits of exercise during pregnancy [33]. Moreover, authors have found that prenatal exercise is safe and beneficial for fetuses.

Maternal exercise is associated with reduced odds of macrosomia and is not associated with neonatal complications or adverse childhood outcomes [3,36,37,38,39,40,41]. In a retrospective population study by Su et al., including all children born in Xiamen, China, in 2011–2018, the data revealed that 6982 (9.37%) of their mothers were obese and 8874 (12.07%) were overweight. Women from both these groups were more likely to develop PTB, completion of pregnancy by caesarean section, and macrosomia [42]. Similar results were obtained by Shahla et al., in a retrospective cohort study in Iran in 2008–2009, who concluded that an increased BMI level increased the risk of an unfavorable course of pregnancy, including premature delivery [43]. In our study, logistic regression showed that the risk of preterm labor increased with higher maternal BMI before pregnancy. An attempt was also made to assess how the pre-pregnancy BMI affects the birth weight of newborns and their Apgar score. The smallest group of participants (both in the group which had the full-term birth and PTB) were women underweight before pregnancy (4.6% of all women in the study). Interestingly, obese or overweight mothers accounted for as much as 30.7% of all studied women from both groups. These data show the scale of the problem of overweight and obesity in the Polish population. A similar distribution of data is presented in the study by Vince et al. [44], whose aim was to determine the relationship between maternal body mass index before pregnancy (pp-BMI) and the course of pregnancy in pregnant women in Croatia in 2017. Among 32,051 pregnant women, 5.3% were underweight, 65.5% had a normal BMI, 20.4% were overweight, and 8.8% were obese. Preterm deliveries occurred more frequently in underweight and obese women (*p* < 0.001) [44].

Importantly, the literature data indicate that prematurity is not directly related to high BMI (>30, obesity), but results from medical complications that usually accompany high BMI [45]. Our analysis shows that the mothers of children who had full term births were more likely to have normal weight or be underweight, while mothers who had preterm births were more likely to be underweight, overweight, or have obesity. In detailed analyses, we noted that that the problem of being underweight in the mother affected children born before 32 weeks of age to a greater extent, while overweight and obesity in the mother affected children born between 32–37 weeks of pregnancy to a greater extent.

One of the most important findings of our study is that pre-pregnancy BMI affected the mode of the childbirth. Women who had normal pre-pregnancy BMI or were underweight had a lower risk of having a cesarean section (CS) compared to women with overweight or obesity. The last two decades have seen an increase in CS worldwide. In the middle and highly developed countries, almost half of the women gave birth by CS. Poland has one of the highest percentages of cesarean sections (42%) in Europe, where the average is 27% [46]. We are still looking for strategies to reduce CS and we should also consider education on the benefits of maintaining a healthy BMI as part of pre-conceptual care. Many authors obtained results that associated increasing BMI with increasing rates of adverse obstetric outcomes, including higher rates of CS [46,47,48].

There were changes in behavioral patterns during the COVID-19 pandemic that were associated with lockdowns and restrictions of access to certain resources. During the lockdown, trends of unfavorable changes were observed: decreased physical activity, increased sedentary time, increased snacking, decreased consumption of fresh food (especially fruit and fish), and increased consumption of sweets, cookies, and cakes. Yet, the opposite trends were also observed: increased home cooking and increased physical activity. All of these trends displayed associations with various individual characteristics [36,37,38]. Although our study was conducted before the COVID-19 pandemic, the results obtained allow for strategies to be developed in case social isolation becomes necessary. It is necessary to maintain positive role models among pregnant women as well as those planning to become pregnant, including offering them education or online exercises [49]. In line with Miranda et al. [32], our results also highlight the need for a multidisciplinary approach during prenatal care to reinforce the adoption of health-promoting behaviors during pregnancy.

The study findings align with recommendations from the American College of Obstetricians and Gynecologists (ACOG), which emphasize the positive impact of physical activity on pregnancy outcomes. Women who reported being physically active before and during pregnancy exhibited a significantly higher likelihood of achieving full-term births, accompanied by a noteworthy reduction in the incidence of very-premature and extremely premature births [50].

Additionally, the Chief Medical Officers’ Physical Activity Guidelines in the UK support the notion that promoting physical activity is integral to overall health, with potential benefits extending to maternal and perinatal outcomes [51]. Importantly, our study identified a correlation between pre-pregnancy body mass index (BMI) and the mode of birth, with a higher BMI associated with an increased rate of cesarean sections. Moreover, pre-pregnancy BMI influenced the week of pregnancy completion, with inappropriate BMI contributing to a rise in the percentage of premature births.

## 5. Strengths and Limitations

The main strength of the study was that the data were collected in multi-centers in Poland, allowing for generalization of our conclusions to the Polish population. The relatively large number of study participants allowed for the subgroups analysis. Medical records used for the data collection were objective and reliable and could not be affected by the subjective assessment of mothers.

From this study we excluded women with multiple pregnancies, recognizing that this condition could limit their physical activity. On the one hand, this assumption provided us with greater group homogeneity and avoided misinterpretation of the data. Multiple pregnancies statistically lead to preterm birth more often and, in consequence, babies with lower birth weight due to their prematurity. These are also variables that can indirectly influence Apgar scores. In addition, multiple pregnancies are mostly terminated by CS [52]. Therefore, in analyses that include both singlet and multiple pregnancies, the impact of physical activity on labor and neonatal outcomes could be attenuated by the negative consequences of multiple pregnancies on these parameters. On the other hand, however, it would be valuable to analyze the outcomes of subgroup of women with multiple pregnancies, as well as women with pregnancy-related complications, such as shortening of the cervix or assisted conception.

In the context of our study, it is noteworthy to address the upper limit for recommended exercise levels during pregnancy. While our findings demonstrate the positive impact of self-perceived exercise before and during pregnancy on various birth outcomes, it is crucial to acknowledge that there exists a need for clear guidelines regarding the upper threshold of physical activity for expectant mothers. The current literature, including recommendations from reputable sources such as the American College of Obstetricians and Gynaecologists (ACOG), underscores the importance of regular physical activity during pregnancy [50]. However, the specific upper limit remains an area that requires precise delineation. Future research and healthcare guidelines should aim to establish evidence-based thresholds for exercise intensity, frequency, and duration to ensure the safety and well-being of both the pregnant woman and the developing fetus. Addressing this aspect is vital for providing comprehensive guidance to healthcare professionals and expectant mothers, fostering a balanced and health-promoting approach to prenatal physical activity.

One of the limitations of our study was that analysis did not include indications for caesarean section. However, it seems that the data obtained may be useful in the future to distinguish the analysis in only CSs performed due to lack of progress of labor, prolonged second period of labor, or threatened asphyxia. Moreover, for the determination of neonatal weight, centile charts for girls and boys were not used to determine percentile values; instead, only birth weight was used, which was then classified into specific birth weight categories. In future analyses, it would be extremely valuable to use a reference to centile charts for this variable.

Another limitation of the study was the lack of information on the type, intensity, duration, and frequency of physical activity undertaken and the length of time for which it was carried out. The data collected was based only on the subjective assessment of the exercise undertaken by the mothers. In future studies it would be valuable to use standardized tools, e.g., Pregnancy Physical Activity Questionnaire [53] or accelerometers to more precisely assess the level of physical activity. To determine the intensity of physical activity, it would be reasonable to use standardized fitness tests (e.g., to establish maximal exercise capacity through the maximal oxygen uptake and maximum heart rate values). Then, the use of heart rate monitors would allow for proper continuous monitoring of the intensity of physical activity [54]. It would also be useful to assess the impact of maternal exercise on the blood pressure values recorded in the pregnancy chart and the pattern of physical activity in the postpartum period (including the time of return to pre-pregnancy weight). These observations should be extended to the assessments of body mass composition and nutritional status. This, in turn, would allow for an analysis of how physical activity influences weight gain during pregnancy and thus how weight gain affects the birth outcomes.

According to the literature, an important issue in assessing the nutritional status of a child’s mother is taking into account the quality of consumed products and identifying deficiencies in the diet. Particularly important is the deficit of elements such as zinc or iron, an inadequate concentration of which in the body may disturb the functioning of the immune system, thus increasing the risk of preterm labor [55]. In our study, most women declared supplementation with complex vitamin preparations. However, we were not able to assess whether or not, and to what extent, these women had vitamin and micro deficiency and macronutrients, and whether or not they were supplemented by appropriately selected preparations.

Since national minorities constitute a marginal part of Poland’s population (around 2%), we did not ask the study participants about their ethnicity. However, due to the increasingly intense migration process in Europe, this issue is worth addressing in future research.

Despite the indicated weaknesses of our work, the obtained results may be useful in planning future, more complex studies aimed at improving a healthy lifestyle before and during pregnancy.

## 6. Conclusions

The study revealed that women who reported being physically active before and during pregnancy were more likely to have full-term birth and had a lower incidence of very premature and extremely premature births. Women who exercised during pregnancy more often gave birth to children with a normal body weight and were less likely to have children with low, very-low, or extremely low body weight. Pre-pregnancy BMI affects the method of pregnancy termination—in the group of women with a higher BMI there were more cesarean sections. Pre-pregnancy BMI also influenced the week of pregnancy termination, i.e., incorrect BMI contributed to an increase in the percentage of premature births.

Our findings indicate that promoting physical activity and weight management remains a priority in public health policy, and women of childbearing age should be encouraged to adopt or maintain an active and healthy lifestyle during pregnancy in order to avoid sedentary- and obesity-associated risks affecting birth and newborns’ health.

## Figures and Tables

**Table 1:** Demographic characteristics of study participants (*n* = 394).

Characteristics	*N*	*%*
Maternal age (years)		
<24	37	9.4%
25–29	118	30.0%
30–34	153	38.8%
>35	86	21.8%
Place of residence		
rural	79	20.1%
City below 20,000 residents	12	3.0%
City 20–100,000 residents	47	11.9%
City 100–200,000 residents	31	7.9%
City with over 200,000 residents	225	57.1%
Education		
Primary school	36	9.1%
High school	89	22.6%
University degree	269	68.3%
Marital status		
Single	52	13.2%
Married	289	73.4%
Cohabiting	53	13.4%

**Table 2:** Maternal data and birth outcomes.

Gestational Age at Delivery (Weeks)		
Extremely preterm (less than 28 weeks)	15	3.8%
Very preterm (28 to 32 weeks)	49	12.4%
Moderate to late preterm (32 to 37 weeks)	89	22.6%
Full-term pregnancy	241	61.2%
Mode of childbirth		
Vaginal birth, VB	207	52.5%
Caesarean section, CS	182	46.2%
Vacuum/forceps delivery	5	1.3%
Maternal exercise before pregnancy		
Yes	133	33.8%
No	261	66.2%
Maternal exercise during pregnancy		
Yes	59	15.0%
No	335	85.0%
Pre-pregnancy BMI category		
Overweight and obesity (25.0 to <30)	121	30.7%
Normal weight (18.5 to 24.9)	255	64.7%
Underweight (<18.5)	18	4.6%
Maternal weight gain [kg]		
<−5; 0)	5	1.3%
<0>	3	0.7%
(0; 7>	48	12.2%
(8; 14>	191	48.5%
(15; 21>	117	29.7%
(22; 32>	30	7.6%
Newborn’s birth weight		
Incredibly low birth weight, ILBW	5	1.3%
Extremely low birth weight, ELBW	15	3.8%
Very-low birth weight, VLBW	28	7.1%
Low birth weight, LBW	55	14.0%
Normal weight, AGA	253	64.2%
Large for gestational age, LGA	38	9.6%
Apgar score in 1st minute		
0–3 points	11	2.8%
4–7 points	47	11.9%
8–10 points	336	85.3%

## Data Availability

Data are contained within the article and supplementary materials.

## Supplementary Materials

The following supporting information can be downloaded at: https://www.mdpi.com/article/10.3390/nu15234894/s1, Table S1: *t*-Student test; maternal exercise before pregnancy. Table S2. Logistic regression for the analyzed variables: group; the mother’s pp weight; mother’s pp-BMI; weight gain in pregnancy. Table S3. Likelihood ratio test for the analyzed variables: group; the weight of the mother at the time of pregnancy; mother’s pp-BMI; weight gain in pregnancy. Table S4. Hosmer-Lemeshow test for the analyzed variables: group; pp mother’s weight; Mother’s pp-BMI; weight gain in pregnancy. Table S5. Model for the analyzed variables: group; the weight of the mother at the time of pregnancy; Mother’s pp-BMI; weight gain in pregnancy. Table S6. Hosmer-Lemeshow division for the analyzed variables: group; the pre-pregnancy weight of the mother; Mother’s pp-BMI; weight gain in pregnancy. Table S7. Exercise before pregnancy and gestational age at delivery [weeks]. Table S8. Exercise started during pregnancy and gestational age at delivery [weeks]. Table S9. Exercise started during pregnancy and newborn birth weight [g]. Table S10. Mode of the childbirth vs. pre-pregnancy BMI category. Table S11. Gestational age at delivery category [weeks] vs maternal pp-BMI.
