# Supplementary material for: Can Pre-Pregnancy Body Mass Index and Maternal Exercise Affect Birth and Neonatal Outcomes—A Cross Sectional Study
Source: Nutrients. 2023 Nov 23;15(23):4894. doi: 10.3390/nu15234894 (PMC10708388; doi:10.3390/nu15234894)
Supplement: Supplementary file 1 [file nutrients-15-04894-s001.zip › questionnaire.pdf]

INFORMATION FOR THE PATIENT AND HIS/HER FAMILY
(information for parents)

Study title:	"Factors predisposing to prematurity, hypotrophy and malformation of the newborn".

The study is conducted by ………………………………………………………………. The scientific work is planned as …………………………. The supervisor of the scientific study is ………………………………………………………………………………………

The study is concerned with finding out the factors predisposing to prematurity, hypotrophy and malformation of the newborn. The study does not interfere with the treatment process, and is only an analysis of data obtained from parents through a questionnaire survey and data contained in medical records. Participation in the study carries no risk.

The study will initially include newborns hospitalised in the ………………………………….
whose parents/guardians give informed consent to participate in the study.

The aim of the study is to determine the relationship of selected social, medical and psychological factors on the incidence of preterm birth. and psychological factors on the incidence of prematurity, hypotrophy and congenital malformations in newborns and to analyse clinical problems in preterm newborns.

All personal data used for the study are processed ………………………., according to the
Art. 6 (1) of Regulation (EU) 2016/679 of the European Parliament and of the Council of 27 April 2016 on the protection of individuals concerning the processing of personal data and the free movement of such data, and repealing Directive 95/46/EC (GDPR), to the extent and for the purpose necessary to conduct the study, as well as for archival and statistical purposes.

All data will be made public in the research paper in an anonymous manner, preventing the identification of individual patients.

Any study participant may withdraw from the study at any time without giving a reason. Withdrawal from participation does not entail any consequences or loss of benefits to which the patient is otherwise entitled. In the absence of sufficient information, the study author may exclude the patient in question from the study, of which the patient's parents will be informed.

If you have any questions, please contact ……………………………………. by e-mail ………………………or by telephone ……………………..

INFORMATION FOR THE PATIENT AND HIS/HER FAMILY
(information for parents)
Programme and principles of the study:

1. Presentation of comprehensive information about the study.
Discuss all aspects of the study with the parents, during a meeting in the Obstetrics, Gynaecology, Neonatal/Premature Intensive Care Unit.

2. Giving informed consent to the parents of the newborn.
Once comprehensive information has been provided and all questions related to the study have been answered, informed consent to participate in the study is given.
Due to the age of the patients (minors), the study is conducted with the consent of the parents (legal guardians).

3 The parent answers the survey questionnaire.

4. Analysis of the information contained in the medical records.

5. Assignment of an identification number in the database.

6. Statistical analysis of the data obtained.

7. Compilation of survey results.
Comparison of the research results with other research conducted in this field in Poland and worldwide.

Research results will be published in scientific journals.

date	signature of the researcher

INFORMED CONSENT TO PARTICIPATE IN THE STUDY
(consent of child's legal guardians)

Study title	"Predisposing factors for prematurity, hypotrophy and neonatal malformations".
Name of the child's mother:	
PESEL* (of the child's mother):<br>*ID number	
Names of the patient's legal guardians:	

We, the undersigned, declare that we have read and understood the 'Information for Patients and their Families' and have received answers to the questions asked to our satisfaction. We know that participation in the study does not affect the treatment of the child and is only an analysis of the information obtained through the questionnaire survey and the data contained in the medical records.

We voluntarily consent to our child's participation in this study and are aware that we can withdraw from the study at any time without stating a reason and that withdrawal from participation does not entail any consequences or loss of benefits to which we are otherwise entitled.

According to the Art. 6 (1) of Regulation (EU) 2016/679 of the European Parliament and of the Council of 27 April 2016 on the protection of individuals concerning the processing of personal data and the free movement of such data, and repealing Directive 95/46/EC (GDPR), we consent to the processing of our child's personal data by ……………………………………., identifying (ID)………………., to the extent and for the purpose necessary to carry out the study, as well as for archival and statistical purposes. We are aware of the voluntary nature of the data provided. We have also been informed that we have the right to access the content of the data concerning our child, to correct, modify and exercise other rights under the above-mentioned Act.
We are aware that in case of any questions or doubts we can contact ……………. by e-mail …………………. and by phone ………………………….

date	signatures of the patient's legal guardians
date	signature of the person taking the consent

Dear Sir or Madam,

The following questionnaire has been prepared to carry out a research study on the risk factors for preterm birth, malformations and fetal hypotrophy.

The results obtained will enable us to improve the quality of care for pregnant patients at high obstetric risk and will also provide insight into the causes of abnormalities in the perinatal period.

All information obtained is anonymous and will be used for research purposes only.

Thank you in advance for taking part in the study.

I. Sociodemographic data

1. Age of the child's mother (at the time of the child's birth) ☐
2. Age of the child's father ☐
3. Place of residence
   a) rural
   b) city below 20,000 residents
   c) city 20-100,000 residents
   d) city 100-200,000 residents
   e) a city with over 200,000 residents
4. Place of residence of the mother
   voivodship :...............................
5. Education
   a) Primary school
   b) High school
   c) University degree
6. Marital status
   a) single
   b) married
   c) cohabiting
7. Occupation of the child's mother

....

8. Occupation of the child's father

....

9. Have you been exposed to any harmful agents in your work/place of residence (other than during your pregnancy)?

   a) No
   b) Yes

10. Has your partner been exposed to harmful agents at work/place of residence?

   a) No
   b) Yes

11. How do you assess your living conditions and economic status?

   a) well
   b) average
   c) bad

II. Course of pregnancy and childbirth

1. The last pregnancy was:

   a) Singleton
   b) Twin/Triple

2. Did you use Assisted Reproductive Technology (ART)?

   a) No
   b) Yes :
   - in vitro fertilisation (write please a protocol: fresh or frozen embryo transfer/natural or stimulate cycle)
   - insemination
   - other

3. Please indicate the week of the last pregnancy termination (number of ending weeks) ☐

4. Please indicate the mode of birth

   a) Vaginal birth, VB
   b) Caesarean section, CS
   c) Vacuum/ forceps delivery

5. What was the reason for the onset of labour/the doctor's decision to terminate the pregnancy (in case of iatrogenic labour?

   a) Regular uterus contraction
   b) Bleeding from the vagina
   c) Premature rupture of membranes (PROM)
   d) Danger of fetal distress (threatened birth asphyxia)
   e) Other (f.i. maternal reasons, health conditions)

6. During your last pregnancy did you experience (add week of pregnancy):

   a) vaginal bleeding
   b) vomiting
   c) headaches
   d) swelling
   e) vaginal discharge

7. Last pregnancy, was it a high-risk obstetric pregnancy? If yes, please specify the reason.

   a) No

b) Yes (choose in the table):

National Institutes of Health: high–risk pregnancy categories* https://www.nichd.nih.gov/health/topics/high–risk/conditioninfo/factors	
Existing health conditions	Hypertensive disorders, polycystic ovarian syndrome, diabetes, renal disease, autoimmune disease, thyroid disease, infertility, obesity, HIV/AIDS
Age	Adolescent
	First–time pregnancy after 35y
Lifestyle factors	Alcohol use
	Tobacco use
	Illicit drug use
Conditions of pregnancy	Multiple gestation
	Gestational diabetes
	preeclampsia and eclampsia

8. Have you had any infections during your pregnancy (vaginal infections/ urinary tract infections/ gastrointestinal tract infections/ respiratory tract infections)?

a) No
b) Yes
- 1st trimester
- 2nd trimester
- 3rd trimester

9. Did you take any medication during your pregnancy?

c) No
d) Yes
- 1st trimester
- 2nd trimester

3rd trimester

10. Did you receive dental treatment before/during your pregnancy?

a) No
b) Yes, only before pregnancy
c) Yes, before and during pregnancy, treatment at ...... weeks of pregnancy
d) Yes, only during pregnancy

If you have been treated by a dentist, please specify what the treatment consisted of.

11. Have there been any preterm births in your family?(degree of relationship

a) No
b) Yes, week of pregnancy termination ☐/☐

12. Did you have a serological examination for toxoplasmosis (IgG, IgM antibodies) during pregnancy?

a) No
b) Yes, at ☐ weeks of pregnancy

13. During pregnancy, did you undergo invasive prenatal diagnosis (amniocentesis, cordocentesis, chorionic villus biopsy)?

a) No
b) Yes (type).......

14. Did you undergo X-ray examinations during your pregnancy?

a) No
b) Yes

15. Was fetal growth retardation (IUGR/ SGA) diagnosed during pregnancy?

a) No
b) Yes

16. During the pregnancy, were any abnormalities found in the foetus during the ultrasound check-up?

a) No
b) Yes:..
....

17. Did you work active during your pregnancy? If no, please go to question 19.

a) No
b) Yes

Were you exposed to any harmful substances during your work? If yes, please specify what these factors were.

a) No
b) Yes
- noise
- high temperature
- radioactive radiation
- ionising radiation
- vibrations
- chemical / biological agents
- other..

18. . Were you exposed to severe work-related stress during your pregnancy?

a) Yes
b) No

19 . Did you supplement your diet with folic acid during pregnancy? Please give the dose, the name of the preparation and the duration of intake

a) Yes (write name and dose if you remember)
b) No

20. Did you supplement your diet with DHA-enriched supplements during pregnancy?

a) Yes
b) I don't know/ remember
c) No

21. Did you do any exercise* before you got pregnant (last 6 months before pregnancy)?*Exercise-all forms of physical activity that were planned, structured, repetitive, and performed with the goal of improving health or fitness, including active transportation, and autonomous and supervised physical exercise, but excluding normal home and work-related activity home activity

a) No
b) Yes

22. Did you do any exercise* during your pregnancy? (additional, new physical activity - excluding activity continued from before pregnancy) *Exercise-all forms of physical activity that were planned, structured, repetitive, and performed with the goal of improving health or fitness, including active transportation, and autonomous and supervised physical exercise, excluding normal home and work-related activity home activity

a) No
b) Yes

23. Did you remain sexually active during your pregnancy?

a) Yes
b) No

24. Do you or anyone close to you smoke cigarettes (if someone it means a person close to you, a family member/a roommate/ a co-worker - longer exposure to passive smoke inhalation)?

a) Yes, I smoke
b) Yes, someone close to me
c) No

25. Did you drink alcohol during your pregnancy?

a) Yes, I did during all pregnancy
b) Yes, I did but I’ve stopped when I’ve got to know that I am pregnant
c) No

If you had your first child, please go to nest section

III History of previous pregnancies and births

1. Please indicate the week of termination of your previous pregnancies
   a) 1st
   b) 2nd
   c) 3rd
2. Mode of your previous pregnancy(s) end (VB/ CS/ vaccum/forceps)
   a) 1st
   b) 2nd
   c) 3rd
3. Was the course of previous pregnancies normal? If no, please describe the type of complications/infections experienced.
   a) Yes
   b) No
4. Please specify the number of:

Children alive __, Children deceased__.
Spontaneous abortions___, Stillbirths__, Artificial abortions __.

IV Health status

1. Do you have any chronic illnesses (diabetes, hypertension, epilepsy, heart disease, hypothyroidism/ hyperthyroidism, kidney disease, etc.)? If yes, please give the name of the disease unit.
   a) No
   b) Yes

2. Does your partner have a chronic illness? If yes, please name the medical condition.
   a) No
   b) Yes

3. Does your family or your partner's family have any inherited genetic diseases?
   a) No
   b) Yes

4. Have you undergone any operations (on minor pelvis) in the past ? If yes, please specify type of surgery.
   a) No
   b) Yes

5. Apart from pregnancy, did you experience any ailments (headaches, dizziness, fainting, digestive or urinary problems)?
   a) No
   b) Yes

6. Have you had a rubella booster vaccination after the age of 20?
   a) No
   b) Yes
   c) I don't know/ remember

7. Please indicate your height ☐

8. Please give your weight before getting pregnant ☐

9. Give your weight gain during pregnancy (pregnancy chart): ☐

V Newborn's outcomes

1 Date of birth of the child:_ _-_ _-_ _ _ _.

2. Sex of the child
   a) Female
   b) Male

3. Foetal age at birth (weeks)

4. Please give the newborn's birth weight and length:
   Weight:☐, length ☐ cm

5. If known to you, please give the number of Apgar points your baby received at birth (1st minute) ☐ points

The next questions relate to newborn babies diagnosed with congenital malformations. If your child has not been diagnosed with a congenital anomaly, please go to next section.

VI Genetic disorders

1. Please indicate the type of congenital malformation found...............................

2. If diagnostic tests have been carried out to confirm the malformation, please specify what they were: ....

3. Age of the child (or gestational age) at the date the malformation was diagnosed:................(or date the malformation was diagnosed).................................

4. Was the defect diagnosed prenatally

a) No
b) Yes: at _ _ week of pregnancy

5. Which prenatal diagnosis methods did you use? In addition to the methods chosen, please indicate the week(s) of pregnancy in which the tests were performed.

a) Ultrasound, at .... weeks
b) amniocentesis .... weeks
c) cordocentesis .... weeks
d) chorionic villus biopsy............ weeks
e) double/triple test (from maternal blood ....weeks

6. Has your child had a baby/fetal karyotype test performed?

a) Not performed
b) Test failed
c) Not known
d) Performed, result unknown
e) Performed, result known
f) Karyotype result: ....

7. is there a history of the same defect or combination of defects in your family? If yes, please specify in whom the defect was found (parents, siblings, other blood relatives?).

a) No,
b) Don't know
c) Yes......................................

8. is there a family history of other malformations or genetically determined diseases? If yes, please specify in whom they occurred and what the defects were.

a) No
b) Yes.....................................

9 Are you and your partner related?

a) No
b) Not known
c) Yes, degree of relationship:

VI Stress scale PSS-10

Sheldon Cohen, Tom Kamarc I Robin Mermelstein<br>**PSS 10**<br>PL-version: Zygfryd Juczyński i Nina Ogińska– Bulik

…………………….. sex ……….. age …………….date

The questions in this scale relate to your thoughts and feelings about your experiences of events in the last month. Each question asks you to indicate - how often you have thought and felt in the given ways. Despite significant similarities, these are different questions and each should be treated separately. It is best to answer each question fairly quickly, choosing the answer that seems most relevant.

For each question, write the appropriate number in the box to the right, as indicated below:

0 = Never

1 = Almost never

2 = Sometimes

3 = Fairly often

4 = Very often

1.In the last month, how often have you been upset because of something that happened unexpectedly? ☐

2. In the last month, how often have you felt that youwere unable to control the important things in your life? ☐

3. In the last month, how often have you felt nervous and "stressed"? ☐

4. In the last month, how often have you felt confident about your ability to handle your personal problems? ☐

5. In the last month, how often have you felt that things were going your way? ☐

6. In the last month, how often have you found that you could not cope with all the things that you had to do? ☐

7. In the last month, how often have you been able to control irritations in your life? ☐

8. In the last month, how often have you felt that you were on top of things? ☐

9. In the last month, how often have you been angered because of things that were outside your control? ☐

10. In the last month, how often have you felt difficulties were piling up so high that you could not overcome them? ☐
