# Supplementary material for: Can Pre-Pregnancy Body Mass Index and Maternal Exercise Affect Birth and Neonatal Outcomes—A Cross Sectional Study
Source: Nutrients. 2023 Nov 23;15(23):4894. doi: 10.3390/nu15234894 (PMC10708388; doi:10.3390/nu15234894)
Supplement: Supplementary file 1 [file nutrients-15-04894-s001.zip › Tables S1-S11.pdf]

Table S1. t-Student test; maternal exercise before pregnancy

	t-Student test; maternal exercise before pregnancy										
Variable	Group 1: Yes										
	Group 2 No										
	Mean	Mean	t Stat	df	P (T<=t)	N	N	SD	SD	Quotient F	p
	Yes	No				yes	no	yes	no	variances	variances
Pre-pregnancy BMI	22.73	23.81	-2.42432	487	0.00785	176	313	4.95879	4.6200	1.152029	0.28118
Gestional weight gain	13.74	12.82	1.66859	487	0.047921	176	313	5.3347	6.0601	1.290458	0.061504

Table S2. Logistic regression for the analyzed variables: group; the mother's pp weight; mother's pp-BMI; weight gain in pregnancy

Variable	Group; the mother's pre-pregnancy weight
Cardinality - Uninterpreted	0
Cardinality - missing data	0
Significance level	0.05
Cardinality	394
The number of variables in the model	3
Number of iterations for convergence	668
The convergence criterion has been achieved	
Cardinality 0 (1)	241
Cardinality 1 (2)	153

Table S3. Likelihood ratio test for the analyzed variables: group; the weight of the mother at the time of pregnancy; mother's pp-BMI; weight gain in pregnancy

Likelihood-ratio test	
Log Likelihood	–324.857818
–2 Log Likelihood	649.715636
Log Likelihood (free)	–334.764208
-2 Log Likelihood (free)	669.528416
Chi-square statistics	19.81278
Degrees of freedom	3
P value	0.000186
AIC - the Akaike criterion	655.715636
AICc - Adjusted Akaike Criterion	655.76574
BIC - Schwarz's Bayesian criterion	668.255686
Pseudo R2	0.029592
R2(Nagelkerke)	0.053589
R2(Cox–Snell)	0.04019

Table S4. Hosmer-Lemeshow test for the analyzed variables: group; pp mother’s weight ; Mother's pp-BMI; weight gain in pregnancy

Hosmer – Lemeshow test	
Chi-square statistics	13.503125
Degrees of freedom	8

Table S5. Model for the analyzed variables: group; the weight of the mother at the time of pregnancy; Mother's pp-BMI; weight gain in pregnancy

Model	free	pp mother’s weigt	pp-BMI	weight gain in pregnancy
factor b	–0.331405	–0.049315	0.176967	–0.046071
error b	0.531551	0.020241	0.060772	0.016668
–95% CI	–1.373226	–0.088988	0.057856	–0.078739
+95% CI	0.710415	–0.009643	0.296077	–0.013404
stat. Wald	0.388714	5.935973	8.479695	7.640486
p-value	0.532976	0.014835	0.003591	0.005707
OR	0.717914	0.951881	1.193591	0.954974
–95% CI	0.253289	0.914857	1.059563	0.924281
+95% CI	2.034835	0.990403	1.344573	0.986686

Table S6. Hosmer-Lemeshow division for the analyzed variables: group; the pre-pregnancy weight of the mother; Mother's pp-BMI; weight gain in pregnancy

[Hosmer–Lemeshow] group	observed[O]	expected [O]	observed [1]	expected [1]
1	33	11.37107	16	5.330248
2	35	14.266856	14	5.655323
3	27	11.95119	20	8.741265
4	26	12.254149	23	10.82875
5	27	13.228702	21	10.327188
6	16	8.171431	33	16.77416
7	21	11.158669	27	14.331651
8	15	8.351869	32	17.927243
9	23	13.962676	25	15.093218
10	16	11.076111	33	23.198232

Table S7. Exercise before pregnancy and gestational age at delivery [weeks]

	Exercise before pregnancy	Gestational age at delivery				total
		Full term	Moderate to late preterm (32 to 37 weeks)	Very preterm (28 to 32 weeks)	Extremely preterm (less than 28 weeks)	
Figure	yes	93	26	12	2	133
% from column		38.6%	29.2%	24.5%	13.3%	
% from rows		70.0%	19.5%	9.0%	1.5%	
% from total		23.6%	6.6%	3.0%	0.5%	33.8%
Figure	no	148	63	37	13	261
% from column		61.4%	70.8%	75.5%	86.7%	
% from rows		56.7%	24.1%	14.2%	5.0%	
% from total		37.6%	16.0%	9.4%	3.3%	66.2%
Figure	total	241	89	49	15	394
% from total		61.2%	22.6%	12.4%	3.8%	
	Chi^2 Pearsona: 7.62877. df=3. p=0.054340					

Table S8. Exercise started during pregnancy and gestational age at delivery [weeks]

	Exercise started during pregnancy	Gestational age at delivery				total
		Full term	Moderate to late preterm (32 to 37 weeks)	Very preterm (28 to 32 weeks)	Extremely preterm (less than 28 weeks)	
Figure	yes	49	7	3	0	59
% from column		20.3%	7.9%	6.3%	0.0%	
% from rows		83.1%	11.9%	5.1%	0.0%	
% from total		12.4%	1.8%	0.8%	0.0%	15.0%
Figure	no	192	82	46	15	335
% from column		79.7%	92.1%	93.9%	100.0%	
% from rows		57.3%	24.5%	13.7%	4.5%	
% from total		48.7%	20.8%	11.7%	3.8%	85.0%
Figure	total	241	89	49	15	394
% from total		61.2%	22.6%	12.4%	3.8%	
	Chi^2 Pearsona: 14.3300. df=3. p=0.002489					

Table S9. Exercise started during pregnancy and newborn birth weight [g]

	Exercise started during pregnancy	Newborn birth weight [g]					total
		LGA-large for gestational age and normal	LBW- low birth weight	ELBW- extremly low birth weight	VLBW- very low birth weight	ILBW- incredibly low birth weight	
Figure	yes	53	3	0	3	0	59
% from column		18.2%	5.5%	0.0%	10.7%	0.0%	
% from rows		89.8%	5.1%	0.0%	5.1%	0.0%	
% from total		13.5%	0.8%	0.0%	0.8%	0.0%	15.0%
Figure	no	238	52	15	25	5	335
% from column		81.8%	94.5%	100.0%	89.3%	100.0%	

% from rows		71.0%	15.5%	4.5%	7.5%	1.5%	
% from total		60.4%	13.2%	3.8%	6.4%	1.3%	85.0%
Figure	total	291	55	15	28	5	394
% from total		73.9%	14.0%	3.8%	7.1%	1.2%	
	Chi^2 Pearsona: 10.2336. df=4. p=0.036672						

Table S10. Mode of the childbirth vs pre-pregnancy BMI category

	BMI -catogory	Birth option			total
		Vaginal birth	Cesarean section	Forceps or vacuum delivery	
Figure	Healthy Weight	144	108	3	255
% from column		69.5%	59.3%	60.0%	
% from rows		56.5%	42.3%	1.2%	
% from total		36.6%	27.4%	0.8%	64.7%
Figure	Overweight and obesity	50	69	2	121
% from column		24.2%	37.9%	40.0%	
% from rows		41.3%	57.0%	1.7%	
% from total		12.7%	17.5%	0.5%	30.7%
Figure	Underweight	13	5	0	18
% from column		6.3%	2.8%	0.0%	
% from rows		72.2%	27.8%	0.0%	
% from total		3.3%	1.3%	0.0%	4.6%
Figure	total	207	182	5	394
% from total		52.5%	46.2%	1.3%	
	Chi^2 Pearsona: 10.5702. df=4. p=0.031844				

Table S11. Gestational age at delivery category [weeks] vs maternal pp-BMI

Mother's pp-BMI	Group				Total	p–value
	Gestational age at delivery- category [weeks]					
	23–27 weeks	28–31 weeks	32–37 weeks	>37 weeks		
underweight	1	3	3	11	18	p =0.03
%	5.5%	16.7%	16.7%	61.1%		
healthy weight	9	28	52	166	255	
%	3.5%	11.0%	20.4%	65.1%		
overweight and obesity	5	18	34	64	121	
%	4.1%	14.9%	28.1%	52.9%		
Overall	15	49	89	241	394	
